# Immune Profiling of Cancer Patients Treated with Immunotherapy: Advances and Challenges

**DOI:** 10.3390/biomedicines6030076

**Published:** 2018-07-02

**Authors:** Lorenzo Pilla, Cristina Maccalli

**Affiliations:** 1 Medical Oncology Unit, San Gerardo Hospital, 20900 Monza, Italy; lopilla@me.com; 2 Clinical Research Center, Division of Translational Medicine, Sidra Medicine, Doha PoBox 26999, Qatar

**Keywords:** immunotherapy, immune monitoring, T cell responses, soluble markers, genomic determinants

## Abstract

The recent advances in immunotherapy and the availability of novel drugs to target the tumor microenvironment have dramatically changed the paradigm of cancer treatment. Nevertheless, a significant proportion of cancer patients are unresponsive or develop resistance to these treatments. With the aim to increase the clinical efficacy of immunotherapy, combinations of agents and standard therapies with complementary actions have been developed mostly on an empirical base, since their mechanisms of actions are not yet fully dissected. The characterization of immune responsiveness and its monitoring along with the treatment of cancer patients with immunotherapy can provide insights into the mechanisms of action of these therapeutic regimens and contribute to the optimization of patients’ stratification and of combination strategies and to the prediction of treatment-related toxicities. Thus far, none of the immunomonitoring strategies has been validated for routine clinical practice. Moreover, it is becoming clear that the genomic and molecular make-up of tumors and of the infiltrating immune system represent important determinants of the clinical responses to immunotherapy. This review provides an overview of different approaches for the immune profiling of cancer patients and discusses their advantages and limitations. Recent advances in genomic-based assays and in the identification of host genomic relationships with immune responses represent promising approaches to identify molecular determinants and biomarkers to improve the clinical efficacy of cancer immunotherapy.

## 1. Introduction

The control of tumor development and growth by the immune system has been shown to be orchestrated by the elimination, equilibrium, and escape phases [1,2]. The interaction between tumor cells and their microenvironment is regulated by a variety of immune cell types and molecular mechanisms playing a determinant role for patients’ clinical outcome [3,4]. Additionally, tumor evolution and progression are accompanied by continuous remodeling of genetic, epigenetic, and metabolic make-up. Advances in the knowledge of cancer immunology have led to an unprecedented clinical development of immunotherapy with, for the first time, a documented improvement for cancer patients’ survival. Immune checkpoint blockade agents targeting either Cytotoxic T Lymphocyte Antigen-4 (CTL-4) or Programmed Cell Death/Ligand-1 (PD-1/PD-L1), that can unleash anti-tumor immune responses, have been approved for the treatment of different solid tumors, e.g., melanoma, lung, head and neck cancer, bladder, and Merkel cell cancer, as well as some hematological malignancies [5]. These drugs showed durable clinical responses also in cancer patients with advanced diseases, changing the paradigm of cancer treatment [5,6,7,8,9]. However, a significant proportion of patients fail to respond or develop resistance to these treatments [9]. This represents the rationale to investigate whether their clinical efficacy can be increased through the combination of either different agents or of standard therapies with immune checkpoint blockade. Many efforts have been dedicated to the development of combinations based on monotherapy encouraging results, such as CTLA-4 and PD-1/L-1 mAbs. Both the Food and Drug Administration (FDA) and the European Medicinal Agency (EMA) approved the combination of CTLA-4 and PD-1 blockade for advanced melanoma. More than 400 clinical trials have been developed based on observational data for combinations of anti-cancer agents, although mechanistic evidences of their synergistic effects are not available [10,11]. Nevertheless, the identification of the molecular landscape of tumors and of the host’s immunological make-up can provide tools to improve cancer patients’ stratification and for managing and predicting any immune checkpoint blockade-associated toxicities. In this review, an overview of the molecular determinants of immune responsiveness in cancer patients and of immunomonitoring approaches will be provided.

It is likely that a deep understanding of the molecular mechanisms regulating the complex interplay between host and tumor microenvironment (TME) through the molecular classification of cancer patients, will guide to the best choice of treatment, sequence, and combination based on.

## 2. Immunomonitoring of Circulating Immune Cells

**Figure 1:**
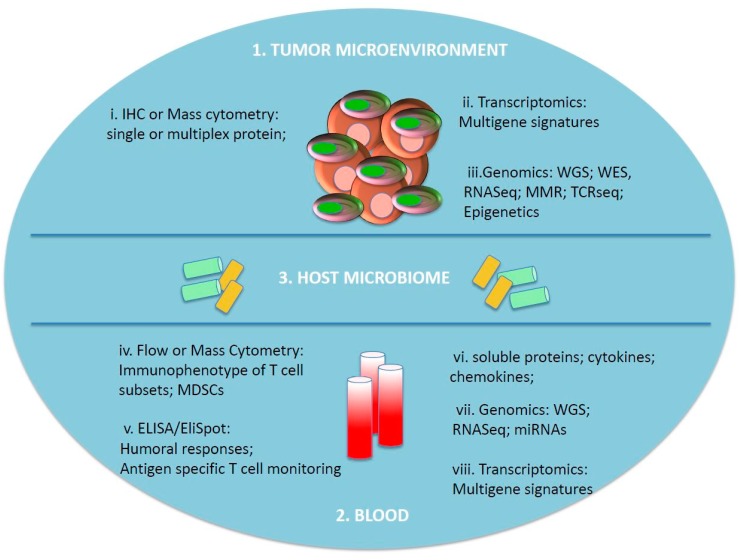
Immune profiling of cancer patients. 1. Tumor and immune cells in tissues specimens can be evaluated through IHC or mass cytometry for: i. defined marker expressions, assessment of type, quantity, and localization of immune infiltration, spatial relationship between tumor and immune cells. ii. Molecular analysis of gene signatures. iii. Genomic and epigenetic analyses. Ideally, a longitudinal analysis of tumor tissues should be performed to monitor changes along with treatment regimens. 2. Peripheral blood represents a minimally invasive procedures to monitor dynamic changes of immune responses through: iv. Immunophenotypic characterization of subpopulations of lymphocytes and monocyte/myeloid cells. v. Monitoring antigen-specific T cell responses or humoral responses. vi. Assessment of soluble biomarkers. vii. Genomic profiling of blood cells. viii. Transcriptomic analyses. 3. Host microbiome can shape the immune responses and affect patients’ clinical outcome after immunotherapy. The assessment of microbiome genomics is assuming a relevant role in immune monitoring of cancer patients. IHC: immunohistochemistry; WGS: whole-genome sequencing; WES: whole-exome sequencing; RNAseq: sequencing of RNA; TCR seq: sequencing of T cell receptor; miRNAs: microRNAs; MDSCs: myeloid-derived suppressor cells; ELISA: enzyme-linked immunosorbent assay; EliSpot: enzyme-linked immunospot assay; miRNAs: micro RNAs.

Major focus has been placed on the identification of the correlation of immune parameters in the peripheral blood of cancer patients treated with immunotherapy. The evidence of increased (≥1000/μL) absolute lymphocyte count (ALC) upon infusion of an immune checkpoint agent represented the first observation of clinical benefit in melanoma patients with advanced disease treated with anti-CTLA-4 mAb [12]. The augmentation of eosinophil count (>100/mm^3^) and of ALC (>1000/mm^3^) in the circulation after the first infusion of anti-CTLA-4 mAb showed correlation with improved overall survival (OS) in a retrospective analysis of *N* = 77 metastatic melanoma patients [13]. In addition, a predictive role of the neutrophils/lymphocytes ratio (N/L) for the clinical efficacy of immune checkpoint blockade was found in a group of metastatic melanoma patients treated with the combination of anti-CTLA-4 mAb and chemotherapy [14]. In this cohort of patients, the baseline *N*/*L* value ≤5 could discriminate patients with statistically significant improved progression-free survival (PFS) from patients with unfavorable clinical outcome [15]. In addition, the modulation of the frequency of T cell subpopulations (Figure 1), in particular, activated central memory or effector memory T cells (CCR7^+^CD45RA^−^ or CCR7^−^CD45RA^−^), has been investigated as a correlative biomarker for immune checkpoint infusions in melanoma patients [12,16,17,18,19]. A deep multiparametric cytofluorimetric analysis of circulating T cells in advanced melanoma patients undergoing the combination treatment with anti-CTLA-4 mAb plus chemotherapy highlighted that increased levels in the peripheral blood of central memory T cells expressing co-stimulatory and activatory molecules (CD45RA^−^CD62L^+^ CCR7^+^ CD27^+^ CD28^+^ BTLA^+^/PD-1^+^) were associated with objective clinical responses [20,21]. Moreover, the same study highlighted that the frequency at baseline of CD3^+^CD4^+^CD45RO^+^BTLA^+^, CD3^+^CD4^+^CD45RO^+^4-1BB^+^, or TH17 T cells could predict patients’ clinical outcome [20,21]. Different studies showed that the frequency in the circulation of CD4^+^ T cells expressing the Inducible T-cell Costimulator (ICOS) molecule was augmented following infusion of CTLA-4 blocking agents in bladder, breast cancer, and mesothelioma patients [22,23,24,25,26]. In some cases, the modulation of ICOS^+^ T cells within few weeks (4–7) following the administration of anti-CTLA-4 mAb was associated with improved OS of cancer patients [22,24,26]. Interestingly, either the frequency at baseline or the modulation in the course of treatment of immune cells endowed with negative immunoregulatory properties, such as T regulatory (Tregs) or Myeloid-Derived Suppressor Cells (MDSCs) have been shown to represent predictors of patients’ clinical outcome for immune checkpoint regimens (Figure 1) [27,28,29,30,31,32]. 

Interestingly, low levels of lactate dehydrogenase (LDH), absolute monocyte and MDSC counts associated with high frequency of Tregs, absolute eosinophil count, and relative lymphocyte count, represented a predictive baseline signature for favorable clinical outcome of melanoma patients treated with anti-CTLA-4 mAb [33].

Tumor cells express antigens, defined as tumor-associated antigens (TAAs), that can be recognized in the form of MHC–peptide complexes by T lymphocytes [34]. The monitoring in the peripheral blood of TAA-specific T cells through the EliSpot assay has been widely exploited to determine the efficacy in terms of immunization of TAA-based cancer vaccines (Figure 1) [35,36]. Interestingly, in some cases, these anti-TAA T cell responses correlated with patients’ clinical outcome [35,36]. Circulating T cells with specific reactivity against TAAs, such as MART-1 and NY-ESO-1, have been observed in melanoma patients administered with CTLA-4 blocking agents [31,37,38,39]. A predictive role for patients’ clinical outcome of baseline detection of T cells recognizing these TAAs has been observed [21].

TAAs are also recognized by antibodies in the context of humoral responses [40]. Indeed, therapeutic interventions with antagonistic mAbs targeting CTLA-4 could augment humoral immune responses against molecularly known TAAs, including NY-ESO-1, and in some cases these responses were associated with patients’ clinical benefit [19,37,39]. All together these results have contributed to show the efficiency of immune checkpoint blockade in unleashing antigen-specific immune responses. Although these investigations have provided insights into the mechanisms of action of immune checkpoint blocking agents, none of the candidate correlative or predictive parameters has been validated as a definitive biomarker in large cohorts of cancer patients.

## 3. Serum Biomarkers

The identification of soluble molecules that could represent predictive biomarkers for immune responsiveness to immunotherapy represents a field of major interest. The monitoring of soluble parameter(s) will allow to utilize relatively simple experimental techniques and easy accessible biological samples, such as serum or plasma (Figure 1). The presence of soluble CD25 (the α-chain receptor of interleukin-2; IL-2) in pre-treatment serum of melanoma patients undergoing anti-CTLA-4 mAb therapeutic regimen has been shown to be an independent indicator of OS [41].

NKG2D ligands (NKG2DLs) represent an indicator of cellular stress and are over-expressed by tumor cells; these molecules bind NKG2D that is either an activatory or a co-stimulatory receptor expressed by NK and T cell, respectively [42]. Shedding of NKG2DLs in the soluble form by tumor cells has been described as part of the tumor escape from immunity through the engagement of the NKG2D receptor on immune cells, resulting in the impairment of their anti-tumor activity [42,43]. Interestingly, soluble NKG2DLs have been detected in the serum of tumor patients with different histological origins with, in some cases, a prognostic role [42,43,44]. The first observation that the clinical activity of the combination of vaccination plus anti-CTLA-4 mAb was affected by soluble NKG2DL was reported by Jinushi et al. [45].

Some years later, it was reported that the baseline serum levels of soluble NKG2DLs (ULBP-1 or -2) could discriminate melanoma patients treated with anti-CTLA-4 mAb plus chemotherapy with improved (median 33.6 months) or poor (median 9.8 or 6.6 months, respectively) OS [21]. Moreover, this study highlighted that the absence of sNKG2DL in the pre-treatment serum of melanoma patients with improved OS correlated with the enrichment of few circulating T cell subsets (e.g., CD3^+^CD4^+^CD45RO^+^BTLA^+^, CD3^+^CD4^+^4-1BB^+^, and Th17) [21]. 

Recently, the role of soluble NKG2DLs as candidate predictive biomarkers of clinical outcome to immunotherapy has been confirmed in a cohort of *N* = 194 melanoma patients treated with anti-CTLA-4 or anti-PD-1 mAb monotherapy or their combinations [46]. The absence of these molecules (MICB and ULBP-1) in the baseline serum was associated with patients’ improved survival (OS = 21.6–25.3 months and *p* = 0.02 and 0.01, respectively), while these molecules were detected in patients with poor survival (OS = 8.8 and 12.1 months, respectively) [46]. The predictive role of sNKG2DLs was independent from the serum levels of LDH, that is a prognostic marker routinely used for patients with a diagnosis of melanoma.

Interleukin-6 (IL-6) and C-reactive protein (CRP) were found as candidate predictive biomarkers for the high-dose IL-2 treatment of patients with metastatic renal cell carcinoma; in particular, high levels of these molecules in the serum (>50 mg/L) were found in patients with progressive disease [47]. CRP is an acute-phase protein of hepatic origin whose levels are commonly increased upon inflammation. CRP is classified as an acute phase reactant detectable in the blood, and its levels are augmented following IL-6 secretion by macrophages and T cells. High levels of CRP (>50 mg/L) at baseline represented an independent predictor of clinical outcome for metastatic melanoma patients treated with high doses of IL-2 [48]. VEGF associated with either fibronectin or CRP in pre-treatment serum was predictive of the clinical outcome of patients treated with high doses of IL-2 or anti-CTLA-4 mAb [49,50]. High levels of LDH (twice upper the limit of healthy donors) could represent a negative predictive marker of clinical response in patients treated with immune checkpoint blockade [51], and decreasing levels of this molecule in the course of treatment were associated with improved OS [30]. Thus far, none of the markers described above are included in the current clinical assessment for treatment decisions.

## 4. Tumor- and TME-Associated Biomarkers

In 2006, Galon and colleagues demonstrated in a seminal paper the predictive role of cytotoxic and memory T cells in determining patients survival [52]. Indeed, patients with a high density of CD3^+^CD45RO^+^ memory T cells in the center and in the periphery of tumors have improved clinical outcome, independently of the T and N stages according to TNM classification. Conversely, a low density of these cells in the TME was associated with very poor survival. The strong predictive value of these parameters was confirmed in subsequent studies [53]. The qualitative, quantitative, and spatial localization of immune infiltrate in colorectal cancer (CRC) [54] has been precisely defined as “immunoscore”, representing a prognostic value with superior significance compared with the American Joint Committee on Cancer (AJCC) and Union for International Cancer Control (UICC) TNM classification [53]. These evidences have led to the classification of cancer in “cold” and “hot” tumors [55].

Further studies showed that also in a specific biologic framework, such as CRC patients with microsatellite-instability, the characterization of the immune infiltrate represents a determinant of tumor recurrence [56]. Recently, Mlecnik et al. [57] demonstrated that, also in the setting of metastatic disease, the type of immune infiltrate correlated with patients’ prognosis. Altogether, these studies highlighted the essential significance of the information derived from the TME to define patients’ prognosis and to predict their sensitivity to specific therapies. However, the limited accessibility to tumor specimens represents the principle hurdle for this type of investigations. In this context, a critical feature is the use of archival versus fresh collected tissues. Archival tissues present some clear advantages, such as the possibility of retrospective analyses of neoplastic tissues avoiding invasive and potentially dangerous clinical interventions. However, these advantages are outweighed by different limitations. The analysis of specific immune cell subsets and genomic profiling in Formalin-fixed Paraffin-embedded (FFPE) tissues is more complex and, in some cases, less reliable compared to the usage of fresh tissues. Additionally, in these types of tissues, the dynamic nature of the immune system cannot be monitored. In this respect, the assessment of PD-L1 in tumors represents a prominent example. The adaptive nature of this molecule and its modulation on the cell surface are regulated by the TME; the dynamic expression of this molecule cannot be monitored in retrospectively collected tissues.

These critical points suggest that is not feasible to grasp the immune system in a single snapshot. Indeed, immune functions are the results of multiple interconnected players which dynamically shape each other. The expression in the TME of PD-L1 might results from the activation by immunotherapy treatment of IFN-γ signaling. Therefore, a longitudinal assessment of immune responses is crucial to understand the complex dynamic evolution of tumor genomic, phenotype, and immunological make-up.

PD-L1 expression in tumor cells and TME can represent a defense mechanism that these cells can use to evade the immune responses. PD-L1 is physiologically expressed by a variety of immune cells in order to restore an immune equilibrium [58]. Since the initial clinical development of anti-PD-1/L1 therapy, the role of PD-L1 expression in tumor and stromal cells has been debated.

Although anti-PD-1 and -L1 antagonistic monoclonal antibodies had a substantial impact in non-small cell lung cancer (NSCLC), only 20% of unselected patients showed clinical benefit to treatment. In most instances, the clinical efficacy of these drugs was registered in patients with PD-L1^+^ tumors. However, durable responses were also observed in patients with tumor cells negative or with low levels of this molecule [59]. Brahmer and colleagues, in the first-in-human study with one anti-PD-1 agent, showed that PD-L1 expression could predict patients’ clinical outcome [60]. Subsequently, several studies in patients with different types of tumors, including NSCLC, melanoma, and renal cell cancer (RCC) have demonstrated the predictive role of PD-L1 expression to identify patients who can benefit from anti PD-L1-targeting therapy.

In 2015, the FDA approved an anti-PD-1 blockade agent for the treatment of NSCLC only for patients with expression of PD-L1 in tumor cells >50%, based on the evidence that these patients had a prolonged progression-free survival and OS compared with patients with lower PD-L1 expression [61,62,63,64]. The complexity and the dynamic nature of tumor–host interactions during cancer development and treatments require a more comprehensive approach for tumor and TME molecular and genomic characterization, in order to evaluate multiple parameters simultaneously (Figure 1).

The investigation of the molecular traits of the TME is crucial also in the case of chemotherapy, such as neoadjuvant chemotherapy [65,66,67]. Denkert and colleagues showed that the levels of stromal tumor-infiltrating lymphocytes (TILs) can represent a predictive marker of clinical responses to neoadjuvant chemotherapy, particularly in carboplatin-containing regimens [65]. Multiple methods exist to characterize the nature and immunological profile of the TME, including Immunohistochemistry (IHC), Whole-Exome Sequencing (WES), proteomics, flow cytometry, and others (Figure 1).

The genomic profiling of tumor tissues can provide useful information about the immunogenicity of cancers [68,69]. It has become clear that in some type of tumors, such as lung cancer, melanoma, and microsatellite-instable (MSI) CRC, the mutational load of the tumor can affect patients’ clinical responses to immune checkpoint blockade [70,71,72,73,74]. Non-synonymous mutations in tumor cells can lead to the expression of mutated antigens and neoantigens that display superior immunogenicity compared to the “self-antigens” shared with normal tissues and can elicit efficient anti-tumor immune responses [75,76]. These mutations may determine the immunosurveillance process promoting tumor elimination by the immune cells through the recognition of new and highly immunogenic tumor-specific antigens [76]. Different evidences showed that either T cell responses against neoantigens or tumor mutational burden are predictive of neoantigen generation and of clinical responses to immunotherapy [70,72,73,74,77,78,79]; however, further investigations are warranted to validate the role of neoantigens as predictive biomarkers to immunotherapy. Clinical studies evaluated the clinical outcome of patients treated with anti-PD-1 mAb, presenting mutations in the mismatch repair machinery or in other enzymes involved in DNA replication and repair, such as the DNA polymerase epsilon gene (*POLE*) and DNA polymerase delta 1 (*POLD1*) gene [77]. These studies confirmed that tumors bearing a high mutational burden can be more susceptible to immune checkpoint treatments [77].

Further mechanisms related to host–tumor interactions and their influence on immune checkpoints resistance have been elucidated through the analysis of a cohort of longitudinal tissues from *N* = 56 melanoma patients treated with anti-CTLA-4 mAb and subsequently, upon progression, with anti-PD-1 mAb. WES and T cell receptor (TCR) sequencing have been performed for tumor lesions, showing that a higher clonality of TCR was predictive of clinical responses to anti-PD-1 mAb. Preliminary results in this context were previously reported by the same group [8]. In addition, the proportion of patients showing clinical benefit from immune checkpoint blockade displayed high tumor mutation burden and low copy number loss [80]. Along this line, the extent of copy number loss correlated with the downmodulation of genes with immune functions [80], suggesting that an integrated signature of mutational load and copy number variation could represent a biomarker for patients’ stratification for immunotherapy.

A recent study demonstrated that bystanders T cells, recognizing different epitopes unrelated to cancer, could be detected among TILs; these T cells displayed a variety of phenotypes resembling TAA-specific T cells but could be distinguished on the basis of the lack of the expression of CD39 [81]. This study also highlighted that the lack of clinical responses to immune checkpoint blockade, although in tumors with high mutational burden such as lung cancer and microsatellite instable CRC, could rely on the relative abundance at the tumor site of bystander CD39^−^ T cells [81]. Thus, CD39-expressing T cells might represent useful predictive biomarkers of immune responsiveness to immunotherapy treatments and clinical outcome.

Novel technologies, such as Whole-Genome Sequencing (WGS), WES, RNA sequencing, and TCR sequencing have been exploited to investigate the relationship between tumor, TME, and immune responsiveness, revealing their advantages for biomarkers discovery. However, future efforts are needed to validate these platforms for clinical treatment decisions. Limitations to the exploitation of these techniques are represented by the relatively large efforts and high amount of time required to perform genomic and molecular characterizations of large cohorts of cancer patients. Another limitation is represented by the requirement of tumor samples, at least at pre-treatment and possibly along treatment, for monitoring reasons, thus excluding a sizeable proportion of cancer patients from these studies.

Another innovative approach is represented by single-cell analysis, that can be performed both at the tumor level and for TCR sequencing, providing an accurate profiling of cellular heterogeneity and its relationship to patients’ clinical outcome. The application of these platforms is still in pre-clinical phase; however, it can be envisioned that the application for the monitoring of cancer patients might occur in the next future [82,83,84,85].

MicroRNA (miRNAs) profiling of tumor tissues could also represent a tool to identify biomarkers associated with patients’ immune responsiveness (Figure 1). The modulation of miRNAs levels was associated with immune-related genes in a cohort of patients treated with anti-CTLA-4 mAb [86].

Epigenetics is another area of growing interest in cancer. Indeed, epigenetic modifications are among the critical mechanisms that regulate and skew gene expression toward a more aggressive cancer phenotype. It has been shown that the modulation of epigenetics in the tumor microenvironment can favorably sensitize the tumor response to immunotherapy [87]. Agents that modulate epigenetics in the TME can determine the upregulation of subclasses of TAA, denominated cancer testis antigens (CTAs), that either are recognized by T lymphocytes or can elicit humoral responses. Thus, the study of epigenetic mechanisms in the TME might contribute to the identification of biomarkers predictive of clinical responsiveness to immunotherapy (Figure 1).

## 5. Host Microbiome

The role of the gut microbiota in determining the fate of immunotherapy was initially analyzed through pre-clinical models by comparing germ-free or antibiotic-treated mice to germ-competent mice treated with CpG oligodeoxynucleotide and anti-IL-10 mAb [88]. Effective immune responses were detectable only in mice with a functional microbiome; moreover, the impairment of the immune responses was dependent on the enrichment of MDSCs and the failure of inflammatory signals [88]. Additionally, chemotherapy-mediated anti-tumor activity was impaired in antibiotic-treated or germ-free animals [88]. Notably, lymphoablating or myeloablating chemotherapy and radiotherapy can damage the gut mucosa causing the transmucosal translocation of commensal bacteria and inducing the augmentation of endotoxin levels, thus increasing the levels of systemic inflammatory cytokines leading to the activation of dendritic cells [89]. This phenomenon mediates adoptive cell therapy clinical activity following chemo- and radiotherapy [89]. 

The gut microbiome can affect patients’ responsiveness to immunotherapy [90]. Indeed, anti-CTLA-4 mAb treatment failed in germ-free or antibiotic-treated sarcoma, melanoma, and CRC mouse models [91]. The therapeutic efficacy of immune checkpoint blockade was restored by the adoptive transfer of specific strains of bacteria [91]. Similarly, the therapeutic mouse model of anti-PD-1 showed that mice harboring distinct microbiota displayed variable tumor growth and response to the treatment [92].

Moreover, different dietary habits and frequent administration of antibiotics and drugs can increase the variability of the microbiota, resulting in different effects on immunotherapy’s outcome. The microbiome role in immune responsiveness needs to be accurately dissected; nevertheless, these analyses should be integrated with analyses of the tumor microenvironment and immune responses in order to create biomarker platforms to predict the clinical outcome of cancer patients treated with immunotherapy or combined treatments (Figure 1).

## 6. Conclusions

The remarkable progress in tumor immunotherapy, either with monotherapies or combinatorial therapies, has dramatically changed the paradigm of cancer treatment. The identification of predictive biomarkers could contribute to achieve efficient patients’ stratification and to design optimal sequencing and scheduling for combinations of treatments. Although a variety of markers, including soluble molecules, lymphocyte subpopulations, immune infiltrate, and genomic determinants, have been isolated as candidate predictive biomarkers of immune responsiveness, thus far, none of them has been validated for routine clinical application. In order to identify valid biomarkers predictive of patients’ clinical outcome, assays that are standardized, reproducible, and available in a large number of laboratories are needed. The advent of high-throughput genomic platforms has provided more efficient tools to investigate the heterogeneity of tumors and TME as well as to identify genomic determinants associated with immune responsiveness. However, the limitations in accessibility and availability of patient’s neoplastic tissues, the choice of archival versus freshly collected tissues, and the need to perform longitudinal monitoring of tissues have to be considered. Moreover, the application of these techniques implies complex analyses and data mining. Along this line, the advent of single-cell genomic platforms allows deep investigations of tumor heterogeneity and of the characterization of immune cell infiltration; however, they are still in an early phase of development, and further efforts are needed to validate their exploitation for biomarker discovery. The heterogeneity and complexity of the host genomic, immunologic, and microbiome landscapes increase the complexity of the identification of determinants of immune responses and clinical efficacy of immunotherapy in cancer patients. Therefore, the development of multiparametric analyses as well as the usage of integrated platforms are critical to achieve a comprehensive monitoring of genomic and immunological biomarkers (Figure 1).
